# 血浆中*CDO1*甲基化在肺癌早期诊断中的作用研究

**DOI:** 10.3779/j.issn.1009-3419.2020.102.20

**Published:** 2020-05-20

**Authors:** 攀 王, 洪林 赵, 睿峰 施, 兴雨 刘, 京豪 刘, 凡 任, 青春 赵, 洪兵 张, 永文 李, 红雨 刘, 军 陈

**Affiliations:** 1 300052 天津, 天津医科大学总医院肺部肿瘤外科 Department of Lung Cancer Surgery, Tianjin Medical University General Hospital, Tianjin 300052, China; 2 300052 天津, 天津市肺癌研究所, 天津市肺癌转移与肿瘤微环境重点实验室 Tianjin Key Laboratory of Lung Cancer Metastasis and Tumor Microenvironment, Tianjin Lung Cancer Institute, Tianjin Medical University General Hospital, Tianjin 300052, China

**Keywords:** 半胱氨酸双加氧酶1, 甲基化, DNA, 肺肿瘤, 血浆, CDO1, Methylation, DNA, Lung neoplasms, Plasma

## Abstract

**背景与目的:**

肺癌的发生率和死亡率常居所有恶性肿瘤的首位, DNA甲基化作为表观遗传学之一参与肿瘤的发生发展过程, *CDO1*作为抑癌基因常在肿瘤发生早期便会发生甲基化改变, 因此本研究旨在探讨*CDO1*甲基化在肺癌早期诊断中的价值。

**方法:**

收集肿瘤患者和健康人群的外周血液样本, 游离DNA通过亚硫酸盐修饰并结合实时荧光定量PCR检测CDO1在外周血中的甲基化水平。

**结果:**

肺癌患者的外周血的基因甲基化水平明显高于肺部良性疾病患者及健康人群。肺癌患者*CDO1*的甲基化水平在性别、淋巴结转移和肿瘤原发灶-淋巴结-转移(tumor-node-metastasis, TNM)分期的分层比较中存在显著性差异(*P* < 0.05)。CDO1对肺癌诊断的灵敏度和特异性分别为52.2%和78.6%。其诊断的整体准确度明显高于应用于临床的肿瘤标志物而且对I期、II期患者的诊断灵敏度表现最好(40.8%, 47.1%)。此外, CDO1可有效增加多项联检中诊断的灵敏性。

**结论:**

检测*CDO1*的甲基化水平对肺癌的早期诊断具有潜在的巨大优势。

## Abstract (en)

**Background and objective:**

The incidence and mortality of lung cancer often rank first in all malignant tumors. DNA methylation, as one of epigenetics, often participates in the development and progression of tumors. *CDO1* as a tumor suppressor gene always undergoes methylation changes early in tumor development. Therefore, this study aims to discuss the value of *CDO1* methylation in the early diagnosis of lung cancer.

**Methods:**

Peripheral blood samples were collected from tumor patients and healthy people. Detection of the methylation level of CDO1 in plasma by sulfite modification and quantitative real-time PCR.

**Results:**

The level of gene methylation in peripheral blood of lung cancer patients was significantly higher than that of benign lung disease patients and healthy people. The methylation level of *CDO1* was significantly different in the stratified comparison of gender, lymph node metastasis and tumor-node-metastasis (TNM) stage (*P* < 0.05). The sensitivity and specificity of CDO1 were 52.2% and 78.6%, respectively. The overall accuracy of the diagnosis was significantly higher than that of the clinical tumor markers, and the sensitivity of CDO1 to stage Ⅰ and Ⅱ patients was the highest (40.8%, 47.1%). In addition, CDO1 could effectively increase the sensitivity of diagnosis in multiple joint examinations.

**Conclusion:**

Detecting the methylation level of CDO1 has a potentially huge advantage for the early diagnosis of lung cancer.

相对于其他恶性肿瘤, 肺癌有着极高的发生率和死亡率且长期居于首位。肺癌患者的不良预后很大程度上在于隐匿性转移扩散的发生, 约50%的患者在确诊时已发生肿瘤转移, 而且获得长期生存的患者多为早期根治性肿瘤切除的无转移的患者, 研究^[1]^表明对Ⅰ期非小细胞肺癌(non-small cell lung cancer, NSCLC)患者进行手术切除, 其5年生存率高达83%, 所以缺乏早期筛查肺癌的敏感性方法常常导致肺癌的高死亡率。因此, 开发出有效的早诊早筛的方法对肺癌患者的诊治工作极其重要。DNA甲基化在肿瘤的早期便会稳定的发生, 而且常常导致抑癌基因的转录失活、原癌基因的表达和印记缺失, 与肿瘤的发生发展以及患者的预后密切相关^[2]^。因此, 对可疑肿瘤患者的基因启动子甲基化水平进行早期筛查和诊断是极具潜力的。

半胱氨酸双加氧酶(cysteine dioxygenase, CDO)是一种对人类健康至关重要的非血红素结构的含铁金属酶蛋白酶, 参与催化有毒性的半胱氨酸生物降解为半胱氨酸亚磺酸, 从而调节体内半胱氨酸的浓度, 可帮助降低细胞中的活性氧(reactive oxygen species, ROS)水平^[3, 4]^。人类半胱氨酸双加氧酶1(cysteine dioxygenase 1, *CDO1*)基因位于5号染色体上, 在晚期肺癌中*CDO1*基因表达常常被抑制^[5]^。研究^[6]^认为CDO1的损耗会增强肿瘤细胞中的氧化应激, 从而诱导肿瘤细胞对ROS产生抵抗和促进肿瘤转移, 因此*CDO1*被认为是关键的肿瘤抑制基因, 可能是癌细胞耐药的一个标志。本研究旨在分析*CDO1*基因在肺癌患者中的甲基化水平, 探讨*CDO1*在肺癌早期诊断中的价值。

## 1 材料与方法

### 1.1 一般资料

收集2018年9月-2019年10月在天津医科大学总医院肺部肿瘤外科的肺部肿瘤患者外周血液样本247例, 其中肺癌患者178例, 良性肺部疾病患者69例; 招募院外健康对照组104例。肺癌患者的病理分型包括腺癌112例、鳞癌29例和小细胞肺癌14例, 其他23例; 根据第8版肺癌肿瘤原发灶-淋巴结-转移(tumor-node-metastasis, TNM)分期分为Ⅰ期71例、Ⅱ期17例、Ⅲ期46例和Ⅳ期44例。具体数据见表 1。本研究通过天津医科大学总医院医学伦理委员会审批通过, 所有入组者均签署知情同意书。

**1:** 351例人群的基本临床信息 Clinical characteristics of all the subjects

Category	*n* (%)
All the subjects (*n*=351)	
Gender	
Male	191 (54.4)
Female	160 (45.6)
Age (yr)	
> 60	152 (43.3)
≤60	199 (56.7)
Types of diseases	
Malignant tumor	178 (50.7)
Benign lung disease	69 (19.7)
Health control	104 (29.6)
Smoking history	
Ever	126 (35.9)
Never	225 (64.1)
Malignant tumor (*n*=178)	
Metastasis	
None	86 (48.3)
Yes	92 (51.7)
TNM stage	
Ⅰ	71 (39.9)
Ⅱ	17 (9.6)
Ⅲ	46 (25.8)
Ⅳ	44 (24.7)
Histology	
AD	112 (62.9)
SCC	29 (16.3)
SCLC	14 (7.9)
Others	23 (12.9)
AD: adenocarcinoma; SCC: squamous cell carcinoma; SCLC: small cell lung cancer; TNM: tumor-node-metastasis.	

### 1.2 主要仪器与试剂

实时荧光定量PCR仪(7500)：美国Applied Biosystems公司; 血浆DNA提取试剂盒：博尔诚(北京)科技有限公司; PCR反应液：博尔诚(北京)科技有限公司; 基因甲基化检测试剂盒：北京博尔诚科技有限公司。

### 1.3 血浆DNA的提取与修饰

所有患者于清晨抽取外周静脉血10 mL, 2, 750 r/min离心12 min, 吸取上清血浆3.5 mL, 使用博尔诚(北京)科技有限公司的甲基化基因检测试剂盒提取血浆游离DNA并进行亚硫酸盐转化以备用。

### 1.4 实时荧光定量PCR

PCR扩增引物由博尔诚(北京)公司设计, CDO1的上游引物5’-GGAGATTTTGCGGGTAC-3’, 下游引物5’-GAAACTCTTAAAAAAACGCGAAAC-3’, 探针5’-CCGAAAAAACCGAAAATATACGCGT-3’。PCR反应体系包括25 μL修饰后的DNA, 11.9 μL的4.2×PCR buffer、1.5 μL的Probe、3 μL的FP、3 μL的RP、4 μL的Block、0.4 μL的ddH_2_O和1.2 μL的Taq酶。在96孔板中每个样本重复2次。反应条件为94 ℃ 20 min, 62 ℃ 5 s, 55.5 ℃ 35 s, 93 ℃ 30 s, 共循环45个周期。在荧光定量PCR仪上进行扩增, *CDO1*的甲基化水平采用2^-△CT^(△CT=Ct_目的基因_-Ct_β-actin_)计算。

### 1.5 统计学方法

数据统计采用SPSS 22.0软件进行统计, 采用Graphpad Prism 8.0.1软件分析作图。检验前行正态分布和方差齐性检验, 非正态分布以中位数(25%, 75%)表示, 计量资料两组间比较采用*Mann-Whitney U*检验, 多组独立样本比较采用*Kruskal-Wallis*检验。采用受试者工作特征(receiver operating characteristics, ROC)曲线和约登指数(Youden index)评价*CDO1*基因甲基化对肺癌的诊断价值, 在多项指标联检中至少1项指标为阳性则判定为阳性, *P* < 0.05表示差异有统计学意义。

## 2 结果

### 2.1 *CDO1*甲基化在不同人群中的比较

首先我们发现*CDO1*的甲基化水平在不同年龄的分层中差异具有统计学意义(*P*=0.004), 但不同性别及吸烟史的分层比较中并无显著性差异(*P*=0.35, *P*=0.733)(表 2)。然后将不同人群中*CDO1*基因的甲基化水平进行比较分析, 结果发现肺癌与良性疾病的甲基化水平存在统计学差异(*P* < 0.001), 肺癌与健康对照人群的甲基化水平同样存在统计学差异(*P* < 0.05), 但是良性疾病和健康对照组基因甲基化水平差异无统计学意义(*P* > 0.05)(图 1)。

**2:** *CDO1*甲基化水平与临床病理特征的关系 The relationship between *CDO1* methylation level and clinicopathological characteristics

Factor	CDO1 relative expression (25%, 75%)	*P*
All the subjects (*n*=351)		
Gender		0.35
Male	0.028 (0.013, 0.440)	
Female	0.032 (0.017, 0.300)	
Age (yr)		0.004
> 60	0.120 (0.014, 1.630)	
≤60	0.029 (0.015, 0.083)	
Types of diseases		< 0.001
Malignant tumor	0.092 (0.016, 1.830)	
Benign lung disease	0.018 (0.011, 0.058)	
Health control	0.028 (0.017, 0.050)	
Smoking history		0.733
Ever	0.029 (0.013, 1.506)	
Never	0.030 (0.016, 0.230)	
Malignant tumor (*n*=178)		
Metastasis		< 0.001
None	0.028 (0.013, 0.230)	
Yes	0.680 (0.027, 7.610)	
TNM stage		< 0.001
Ⅰ	0.0280 (0.013, 0.230)	
Ⅱ	0.037 (0.016, 2.910)	
Ⅲ	0.054 (0.014, 2.760)	
Ⅳ	2.350 (0.150, 12.840)	
Histology^a^		0.08
AD	0.058 (0.014, 0.77)	
SCC	0.160 (0.018, 6.210)	
SCLC	2.100 (0.012, 24.940)	
^a^: Because the number of the patients with other pathological types was fewer, the related test was not done.		

**1:**
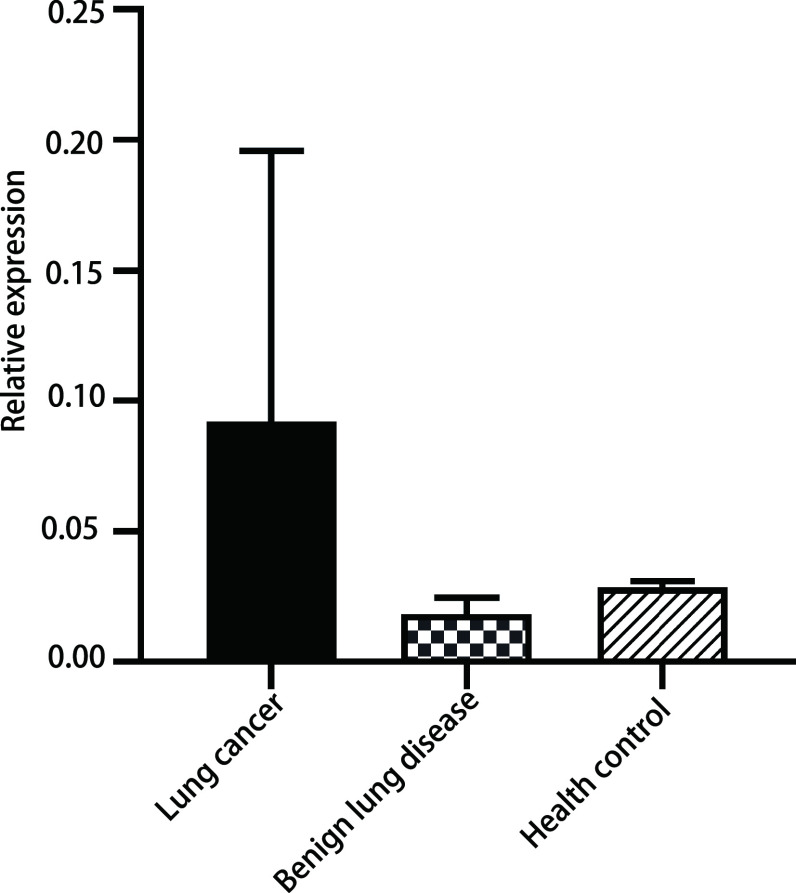
*CDO1*基因在不同分组中的甲基化水平的比较。肺癌组*vs*肺部良性疾病组, *P* < 0.001;肺癌组*vs*健康对照组, *P* < 0.05;肺部良性疾病组*vs*健康对照组, *P* > 0.05。 Comparison of methylation levels of *CDO1* gene in different groups. Lung cancer group *vs* Benign lung disease group, *P* < 0.001; Lung cancer group *vs* Health control group, *P* < 0.05; Benign lung disease group *vs* Health control group, *P* > 0.05.

### 2.2 肺癌患者*CDO1*甲基化与临床病理的关系

我们进一步分析了肺癌患者中*CDO1*的甲基化水平与临床病理的关系, 研究发现在是否有淋巴结转移和TNM分期的分层比较中差异均有统计学意义(均*P* < 0.001), 而且有淋巴结转移的患者和晚期患者其*CDO1*甲基化水平明显高于无淋巴结转移和早期肿瘤患者。此外, 不同病理类型的肺癌患者其*CDO1*基因甲基化水平无明显差异(*P*=0.08)(表 2)。

### 2.3 *CDO1*单基因检测和多基因联检在肺癌中的诊断价值

为了评价*CDO1*甲基化在肺癌中的诊断价值, 首先采用约登指数最大法得到*CDO1*基因的cut-off值, 其中CDO1的约登指数为0.308, cut-off值为0.079。再以ROC曲线下的面积(area under roc curve, AUC)进一步评估。我们通过灵敏度、特异性、准确度以及AUC进行综合比较分析, 在针对单个诊断指标的分析比较时发现CDO1诊断的灵敏度为52.2%, 特异性为78.6%, 准确度为65.2%。在与其余5种临床常用的诊断指标包括癌胚抗原(carcinoembryonic antigen, CEA)、细胞角蛋白19片段(cytokeratin 19 fragment, CYFR21-1)、鳞癌抗原(squamous cell carcinoma antigen, SCC)、胃泌素释放肽前体(pro-gastrin releasing peptide, ProGRP)、神经元特异性烯醇化酶(neuron specific endolase, NSE)进行对比, 结果显示CDO1的灵敏度最高(52.2%), ProGRP的诊断特异性最高(100.0%), CDO1的诊断准确度最高(65.2%)。CYFR211的AUC最大(0.674)。5种临床常用诊断指标的特异性均较高分别为95.0%、96.9%、93.5%、100.0%、81.0%, 但其灵敏度均较低分别为29.0%、37.7%、17.1%、13.7%、37.0%。在进行的多项联合检测中发现, 临床中经典的5项诊断指标的联合检测的灵敏度为69.1%, 特异性为69.4%, 准确度为69.2%, 曲线下面积为0.691。最后将*CDO1*基因加入联检指标结果发现肺癌检测的灵敏度升高到79.2%, 特异性为70.5%, 整体的准确度为74.9%, AUC为0.749(表 3)。

**3:** 单个和多个基因联合诊断效能的分析 The diagnostic efficacy of single and multiple genes combined

Diagnosis index	Sensitivity (%)	Specificity (%)	Accuracy (%)	AUC
CDO1	52.2	78.6	65.2	0.635
CEA	29.0	95.0	46.0	0.623
CYFR211	37.7	96.9	53.2	0.674
SCC	17.1	93.5	37.1	0.551
ProGRP	13.7	100.0	36.3	0.569
NSE	37.0	81.0	48.7	0.587
Five combined check^a^	69.1	69.4	69.2	0.691
Six combined check^b^	79.2	70.5	74.9	0.749
^a^: Combined diagnosis of CEA, CYFR211, SCC, ProGRP and NSE; ^b^: Combined diagnosis of CDO1, CEA, CYFR211, SCC, ProGRP and NSE. CEA: carcinoembryonic antigen; CYFR21-1: cytokeratin 19 fragment; SCC: squamous cell carcinoma antigen; ProGRP: pro-gastrin releasing peptide; NSE: neuron specific endolase; AUC: area under the curve; COD1: cysteine dioxygenase 1.				

为了进一步了解各项肿瘤标志物指标在不同临床分期中的特点, 我们将临床分期共分为Ⅰ期、Ⅱ期、Ⅲ期和Ⅳ期, 并分别比较不同肿瘤标志物检测的灵敏度。通过分析可以发现对Ⅰ期、Ⅱ期和Ⅳ期患者的诊断灵敏度最高的都是CDO1, 其灵敏度分别为40.8%、47.1%和81.8%, 对Ⅲ期患者诊断灵敏度最高的是CYFR211(52.2%)。通过对比发现多项联检能明显整体提高诊断的灵敏度, 临床常用的5项指标联检的灵敏度分别为44.9%、68.8%、87%和88.6%, 而加入*CDO1*的甲基化诊断指标后的6项联检让诊断的灵敏度整体有了更大的提高, 其诊断的灵敏度分别为62.0%、82.4%、87%和97.7%(表 4)。

**4:** 不同临床分期中基因诊断准确度的比较 Comparison of the accuracy of gene diagnosis in different clinical stages

Diagnosis index	Ⅰ (*n*=71)	Ⅱ (*n*=17)	Ⅲ (*n*=46)	Ⅳ (*n*=44)
CDO1	40.8%	47.1%	43.5%	81.8%
CEA	17.1%	18.8%	34.8%	45.5%
CYFR211	14.5%	25.0%	52.2%	63.3%
SCC	4.3%	31.3%	21.7%	27.3%
ProGRP	2.9%	25.0%	21.7%	18.2%
NSE	15.9%	40.0%	46.7%	59.1%
Five combined check^a^	44.9%	68.8%	87.0%	88.6%
Six combined check^b^	62.0%	82.4%	87.0%	97.7%
^a^: Combined diagnosis of CEA, CYFR211, SCC, ProGRP and NSE; ^b^: Combined diagnosis of CDO1, CEA, CYFR211, SCC, ProGRP and NSE.				

## 3 讨论

肺癌是全球第一大癌症, 也是与癌症相关死亡的主要原因^[7]^。肺癌患者预后较差与多种因素有关, 其中之一就是缺乏持久有效的治疗方案, 更重要的因素是诊断时多为晚期, 因为很多肺癌患者在疾病进展到晚期才因典型的临床症状被发现, 因此患者整体预后较差^[8]^。据报道仅有13%的肺癌患者生存时间超过5年, 其死亡率与发病率的比值为0.87^[9]^。

美国国家肺部筛查实验的结果显示, 使用低剂量计算机断层扫描(computed tomography, CT)筛查可将肺癌的死亡率降低20%, 但是这种生存优势是以检测许多性质不确定的肺结节为代价的, 而且其总的假阳性率为96.4%^[10]^, 诊断特异性较差因此很可能需要进一步的随访或侵入性的操作, 而且分析发现良性疾病的手术切除率仍然过高(6%-38%)^[11]^, 此外, 研究表明大约有50%的可疑肺癌患者首次使用侵入性分析方法(经皮穿刺活检或支气管活检)无法确诊^[12, 13]^。因此, 迫切需要新的微创的生物标志物来提高早期诊断的准确性, 减少不必要的创伤性的诊疗并改善预后。以DNA甲基化为主的表观遗传学生物标志物已成为提高癌症诊断最有前途的方法之一。DNA甲基化是一种共价改变, 非常稳定而且常发生于癌变早期。肿瘤特异性的甲基化表观遗传学改变能够频繁在患者血浆DNA中发现, 例如抑癌基因启动子序列的高甲基化水平^[14]^。此外, 即使在肿瘤纯度低的样本中, 也可以通过多种灵敏却经济高效的技术进行检测DNA甲基化^[15]^。因此, DNA甲基化检测在肺癌的早期诊断中较大的潜在优势, 本研究旨在探索*CDO1*在肺癌早诊中的价值。

首先我们对*CDO1*的甲基化水平与人群特征的关系进行分析, 因为有研究表明吸烟、性别、酒精摄入等危险因素对不同基因的甲基化水平有一定的影响^[16, 17]^, Gao等^[18]^对肺癌75个候选基因对应的2, 854个位点进行进行甲基化分析, 结果发现有13个位点与吸烟显著相关而且大部分与低甲基化相关, 仅有cg17928584(*STK32A*)和cg19696491(*CHRNA5*)表现为吸烟诱导的高甲基化。我们对患者性别、年龄、吸烟史等进行分析发现*CDO1*基因在不同性别和吸烟史的组别中启动子甲基化水平差异无统计学意义(*P*=0.35, *P*=0.73), 然而在不同年龄的患者中差异具有统计学意义(*P*=0.004), 既往研究^[19]^表明正常乳腺细胞表现出与年龄相关的甲基化模式而且甲基化位点随着年龄增加而增加, 并且与年龄相关位点在肿瘤与正常组织间存在明显的甲基化差异从而表明与年龄相关的甲基化可能会增加肿瘤发生的风险。多项研究^[20, 21]^也表明年龄的增加会干扰甲基的放置或移除从而对表观遗传基因组产生深远的影响, 而且在组织和血液中均可发现这种年龄相关的甲基化模式。因此我们认为这种模式可能也存在于*CDO1*基因。此外, 对CDO1在肺癌组、良性肿瘤组和健康对照组进行分组研究表明, *CDO1*的甲基化水平明显高于其他两组且差异具有统计学意义(*P* < 0.05)且良性疾病组与健康对照组间无统计学差异(*P* > 0.05), 这表明相比于非肿瘤患者, 基因甲基化水平在肺癌患者中存在明显的差异性甲基化从而有利于肺癌患者的诊断, 因此为其在肺癌中的应用提供了理论支持。

既往研究^[22, 23]^表明临床运用的肿瘤标记物在不同病理类型中有着显著性的差异, 其中NSE在小细胞肺癌(small cell lung cancer, SCLC)中敏感性最高, CYFR211和CEA在NSCLC中敏感性最高, SCC在NSCLC的浓度明显高于SCLC, 而且腺癌中CEA和CA125显著高于鳞癌。因此, 考虑到CDO1是否在特定的肺癌组织学类型中也存在明显的差异, 我们也进行了相关的比较, 研究结果显示*CDO1*在AD、SCC和SCLC中的甲基化水平无统计学差异(*P*=0.08), 这与之前的研究相一致^[15]^, 这也表示*CDO1*的甲基化在肺癌中无组织学差异, 具有较好的一致性, 因此对肺癌的诊断有较好的准确度。

一项单中心的研究对805例可疑肺癌患者的血清SCC、CEA、CYFR211、NSE四种肿瘤标志物的含量进行检测^[22]^, 结果显示诊断灵敏度最高的是CYFR211(24.13%), 其余指标SCC、CEA和NSE的灵敏度分别为7.44%、17.15%和5%。4项指标在特异性方面表现均较好(96.6%-100%)。我们的研究结果也显示在经典的肿瘤5项指标中CYFR211的诊断灵敏性最高(37.7%)并且诊断的准确度也是最高(53.2%), 诊断特异性最高的是ProGRP为100%。临床检测的5项肺癌肿瘤标志物另外的一个特点就是在早期肺癌患者中表达含量较低, 从而导致诊断的灵敏度较低, 这与本实验的研究结果也是相符的。本研究对外周血进行甲基化检测的结果显示CDO1诊断的灵敏度明显高于临床常用的诊断指标(52.2%), 诊断的特异性也较为理想, 为78.6%, 诊断的准确度也明显高于其他5项指标(65.2%), 因此我们认为*CDO1*的甲基化检测能较好地补充临床指标在肺癌诊断中的不足。值得注意的是当多个基因进行联检是诊断的灵敏度会有较大的提高, 但与此同时其特异性也会相应的降低, 因此进行联检时的基因越多并不能代表整体的诊断效果较好, 如何在这两者间找到最佳平衡点仍是需要认真思考的问题。

在进一步的研究中发现, 对I期患者的诊断中传统指标的准确度明显偏低(2.9%-17.1%), 其原因就在于早期相关基因的表达量较低。而基因*CDO1*的诊断准确度却高达40.8%, 也再次验证DNA甲基化在恶性肿瘤中发生较早且利于早期诊断的观点^[24-26]^。而且我们的研究结果显示有淋巴结转移或临床分期较晚的患者其*CDO1*基因甲基化水平也明显高于非淋巴结转移和早期患者且差异具有统计学意义(均*P* < 0.001), 因此DNA甲基化同样存在随着肿瘤的进展其甲基化水平逐渐升高的特点, 这也与既往研究相一致^[27, 28]^。所以随着肺癌患者临床分期的升高, 各项指标的诊断准确性整体有了较大的提高, 不难发现*CDO1*基因不论是早期还是中晚期其诊断的准确度都明显高于传统指标。所以肿瘤患者早期基因启动子甲基化的稳定出现在肿瘤筛查和早期诊断中具有十分巨大的潜在优势, 而且令人欣喜的是目前已有多种DNA甲基化检测试剂盒准备用于临床癌症的诊断, 其中基因*SEPT9*、*NDRG4*和*BMP3*被推荐用于结直肠癌的诊断^[29, 30]^, 肺癌中*SHOX2*基因甲基化在血浆、痰液、胸腔积液的诊断中具有高度的敏感性和特异性而且其诊断试剂盒已经在欧洲用于临床辅助诊断^[31, 32]^, 此外, SHOX2还被认为是预测NSCLC疾病进展的独立生物学标志物^[33]^。

综上所述, *CDO1*基因在肺癌中存在明显的异常甲基化, 在肺癌的诊断中相比于临床应用的肺部肿瘤标志物具有较好的灵敏度和特异性而且在肺癌的早期诊断中具有巨大的潜在优势, 因此采用外周血基因甲基化的检测方法可能为肺癌的早期诊断提供全新的思路和机会。
